# Association between laser flare photometry and symptom duration in primary rhegmatogenous retinal detachment

**DOI:** 10.1007/s10792-022-02532-x

**Published:** 2022-10-05

**Authors:** Leonie Menghesha, Verena Schoeneberger, Stefanie Gerlach, Julia Lemke, Tim U. Krohne, Nicolas Feltgen, Friederike Schaub

**Affiliations:** 1 Department of Ophthalmology, Faculty of Medicine and University Hospital Cologne, University of Cologne, Kerpener Strasse 62, 50937 Cologne, Germany; 2 Department of Ophthalmology, University Hospital Goettingen, Goettingen, Germany; 3 Department of Ophthalmology, University Medical Center Rostock, University of Rostock, Rostock, Germany

**Keywords:** Rhegmatogenous retinal detachment, Laser flare photometry, Blood-ocular barrier disruption

## Abstract

**Purpose:**

The purpose of this study was to investigate preoperative blood-ocular barrier disruption via laser flare photometry (LFP) in patients diagnosed with rhegmatogenous retinal detachment (RRD), and to analyse possible associations with symptom duration and anatomical parameters.

**Methods:**

We retrospectively analysed consecutive patients presenting with RRD at a single centre between January 2016 and March 2020. LFP was performed in both eyes after pupillary dilatation prior to RRD surgery. Symptom duration, extent of retinal detachment, and lens status were assessed. For statistical analysis, we carried out the unequal variances *t* test and Welch’s analysis of variance (ANOVA).

**Results:**

We included 373 eyes of 373 patients (mean age 63.96 years ± 10.29; female:male ratio 1:1.8). LFP values quantified in photon count per millisecond (pc/ms) increased with longer symptom duration when comparing patients with a symptom duration of 0–3 days (*n* = 158; 9.25 ± 6.21 pc/ms) and ≥ 4 days (*n* = 215; 11.97 ± 11.58 pc/ms; *p* = 0.004). LFP values also rose with the number of retinal quadrants affected by RRD (1 quadrant, 6.82 ± 4.08 pc/ms; 2 quadrants, 10.08 ± 7.28 pc/ms; 3 quadrants, 12.79 ± 7.9 pc/ms; 4 quadrants, 31.57 ± 21.27 pc/ms; *p* < 0.001), macula off status (macula on, 8.89 ± 6.75 pc/ms; macula off, 12.65 ± 11.66 pc/ms; *p* < 0.001), and pseudophakic lens status (pseudophakia, 12.86 ± 9.52 pc/ms; phakia: 9.31 ± 9.67 pc/ms; *p* < 0.001).

**Conclusion:**

In RRD patients, blood-ocular barrier disruption quantified by LFP is associated with the duration of symptoms and the disease’s anatomical extent. These results warrant further investigation of the potential clinical use of LFP in RRD.

## Introduction

A disruption in the blood-ocular barrier occurs in response to intraocular inflammation. Slit lamp bio-microscopy is traditionally applied to evaluate an increase in protein in the eye’s anterior chamber, which produces an optical phenomenon called the Tyndall effect, or flare. Laser flare photometry (LFP) already qualifies as an objective quantitative method to noninvasively measure protein particles in the anterior chamber in the photon count per millisecond (pc/ms).

Until now, few studies have investigated LFP values in patients with rhegmatogenous retinal detachment. Hoshi et al. [4] evaluated aqueous flare levels with LFP in eyes with RRD over time after vitrectomy, whereas Chalam et al. [1] investigated them subjectively via slit lamp bio-microscopy. Furthermore, studies by Hoerster et al. and Schroeder et al. demonstrated that laser flare levels before surgery are a predictor when assessing the risk for proliferative vitreoretinopathy (PVR) associated with elevated re-detachment rates in eyes suffering primary RRD [3, 12]. A prospective study by Mulder et al. failed to validate these findings in an independent prospective study. Yet LFP values seem to have a predictive value regarding the postoperative PVR risk. As a consequence, the value of preoperative laser flare values as a predictor for PVR remains unclear [10, 11]. The complex problem of PVR development and the associated rate of retinal re-detachment after primary surgical success is currently the subject of intensive research efforts with the aim to find preventive and therapeutic options. In relation to this problem, the search for influencing factors is also being pursued.

So far, to the best of our knowledge, there is no data describing possible associations between anatomical parameters and symptom duration in eyes with RRD and laser flare values. Therefore, the aim of the present study was to complement previous knowledge by assessing, for the first time, an association between preoperative blood-ocular barrier disruption measured via LFP in terms of symptom duration and anatomic extent in patients with RRD.

## Methods

This single-centre retrospective study relied on data acquired at the Department of Ophthalmology, University Hospital of Cologne, Germany between 27th January 2016 and 12th March 2020. It was approved by the Ethics Committee of the University of Cologne (20-1043). All tenets of the declaration of Helsinki have been followed.

### Collection of clinical data, inclusion, and exclusion criteria

Medical records of 538 consecutive patients (mean age 64 ± 10.3 years) with RRD were reviewed. Epidemiological data (age, gender) and symptom duration (period in days from first recognised symptoms to the day the laser flare value was measured) were taken from the patient file. Ophthalmological examinations included best spectacle-corrected visual acuity (BSCVA) and slit lamp bio-microscopy as well as fundoscopy of both eyes in medical mydriasis with 0.5% tropicamide or 5% phenylephrine hydrochloride. The extent of RRD including affected retinal quadrants (1–4), macular attachment status (macula on, macula off) based on the fundus examination and fundus drawings on the admission day, and lens status (phakia, pseudophakia) were documented. The charts of the patients were reviewed for previous intraocular surgeries and concomitant diseases. Our exclusion criteria were inaccurate laser flare values with standard deviation (SD) of LFP values higher than 15 pc/ms (*n* = 13), unknown symptom duration (*n* = 20), missing preoperative flare measurements (*n* = 57), diabetic macular oedema (*n* = 1), secondary RD including visible proliferative vitreoretinopathy (*n* = 53), history of refractive surgery of the cornea (*n* = 11), cystoid macular oedema (*n* = 1), cornea guttata (*n* = 5), active or inactive uveitis (*n* = 2), proliferative diabetic retinopathy (*n* = 1) and synchisis scintillas (*n* = 1). No eyes with a history of trauma were included. Thus, a total of 165 patients had to be excluded, and 373 qualified for further analysis.

### Laser flare photometry (LFP)

Preoperative LFP of both eyes was performed in medical mydriasis on the day of admission to the department with the Kowa FM-500 Laser Flare-Cell Meter (Kowa Company Ltd, Tokyo, Japan—distributor: Kowa Optimed Deutschland GmbH, Düsseldorf, Germany) to measure the laser flare value in both eyes’ anterior chambers taking at least seven measurements. After excluding the highest and lowest laser flare levels, the average and SD were calculated. To avoid external scattering, the room was darkened during measurements. LFP was performed prior to Goldman three mirror contact lens examination and Goldman applanation tonometry.

### Statistical analyses

Descriptive data were collected and analysed by SPSS (version 25.0 for windows; SPSS, Inc., Chicago, IL). The BSCVA was converted to the logarithm of Minimum Angle of Resolution (logMAR). The following groups were classified according to symptom duration: group 1: 0–3 days (*n* = 158), group 2: ≥ 4 days (*n* = 215), group 2a: 4–7 days (*n* = 154), and group 2b: > 7 days (*n* = 61). Depending on normal distribution of the interval-scaled parameters we had analysed, we conducted the unequal variances *t* test and Welch’s analysis of variance (ANOVA) including Games-Howell post hoc analysis and multiple linear regression for statistical analysis. We observed no homogeneity of variance (Levene’s test, *p* < 0.001). The level of significance was defined as *p* < 0.05.

## Results

Totally 373 eyes with primary RRD were included (right eye *n* = 201, left eye *n* = 172) of 373 patients (mean age 63.96 years ± 10.29; female:male ratio 1:1.8). The distribution of documented patient characteristics and laser flare values is illustrated in Table 1. In patients from whom we had LFP measurements in both eyes (*n* = 356), the mean LFP values were higher (10.98 pc/ms ± 9.94) than in the unaffected fellow eyes (6.68 pc/ms ± 4.53; *p* < 0.001, paired *t* test). A normal healthy eye shows values between 1 and 9 pc/ms. There were no significant differences in laser flare values between men and women. We noted that laser flare values rose significantly in conjunction with macula-off status, in patients with pseudophakia, and with the number of quadrants affected. There was no homogeneity of variance (Levene’s test, *p* < 0.001). Therefore, Welch’s ANOVA was assessed to analyse the extent of RRD (Welch’s F (3, 72.22) = 21.94, *p* < 0.001) (Table 1).

**Table 1:** Epidemiological data and anatomical details on patient cohort

Epidemiological data	Frequency (percentage %)	Laser flare level (mean ± SD in pc/ms)	*p* value	95% Confidence interval (CI)
*Sex*			0.435	[− 1.28, 2.96]
Male Female	241 (64.6) 132 (35.4)	11.11 (± 9.5) 10.27 (± 10.16)		
*Macula status*			< 0.001	[− 5.7, − 1.83]
On Off	182 (48.8) 191 (51.2)	8.9 (± 6.8) 12.7 (± 11.7)		
*Extent of RD*			< 0.001	[9.82, 11.80]
1 quadrant 2 quadrants 3 quadrants 4 quadrants	100 (26.8) 197 (52.8) 55 (14.7) 21 (5.6)	6.82 (± 4.08) 10.08 (± 7.28) 12.79 (± 7.9) 31.57 (± 21.27)		
*Lens status*			< 0.001	[− 5.52, − 1.57]
Phakia Pseudophakia	215 (57.6) 158 (42.4)	9.31 (± 9.67) 12.86 (± 9.52)		

Unequal variances *t* test (sex, macula status, lens status) and Welch’s ANOVA (extent of RRD)

**Fig. 1:**
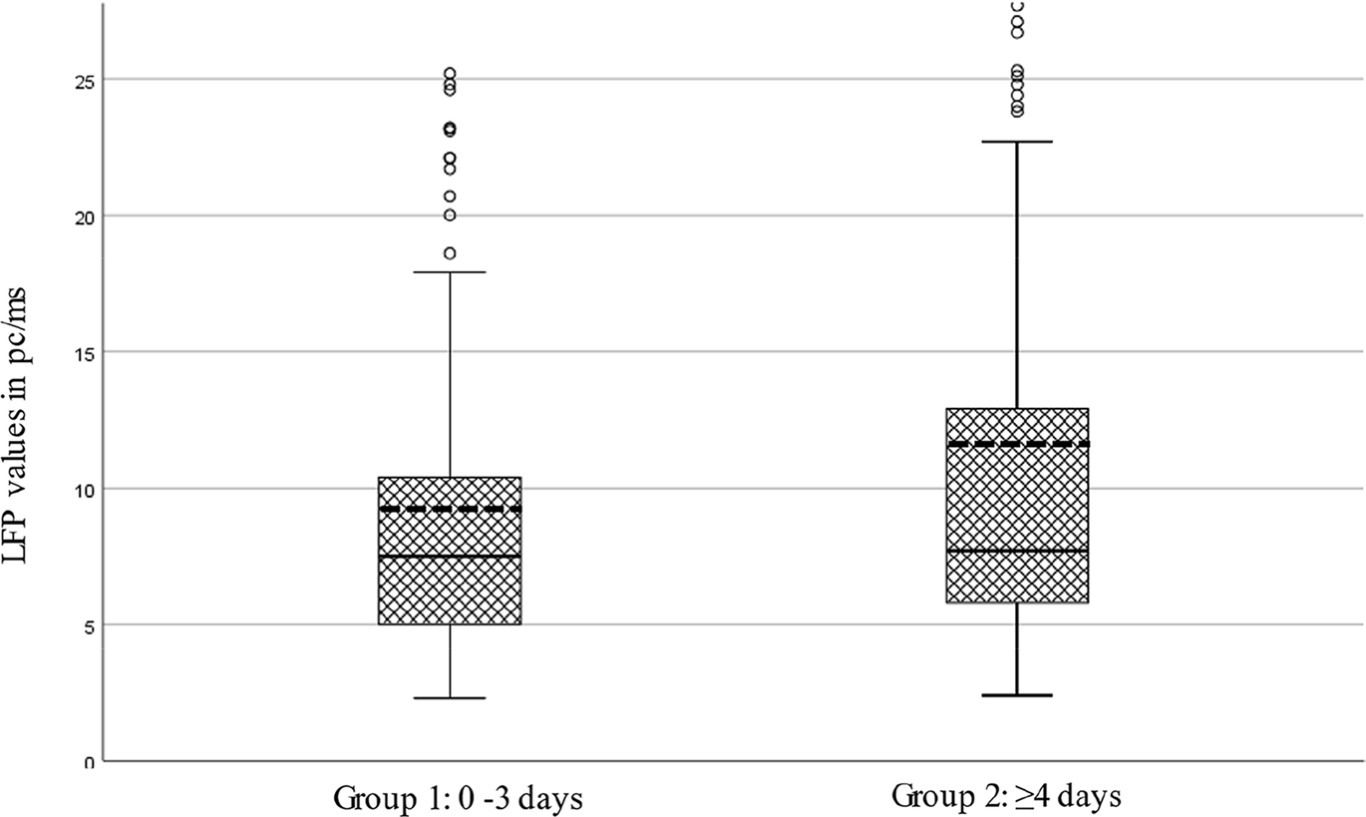
Boxplots for LFP values in pc/ms for symptom duration group 1 (*n* = 158) and group 2 (*n* = 215), *p* = 0.004. Boxes represent first quartile, median (solid lines), mean (dashed lines) and third quartile values; whiskers represent extreme values

Of 373 eyes with RRD, we classified two main groups and two subgroups based on symptom duration. Laser flare values increased significantly with longer symptom duration in groups 1 and 2, as well as in subgroup analysis of group 1 and subgroup 2a (Table 2, Fig. 1).

**Table 2:** Distribution in groups and subgroups. Association with laser flare values

Symptom duration	Frequency (percentage %)	Laser flare value (mean ± SD) in pc/ms	*p* value	95% Confidence interval (CI)
Group 1: 0–3 days	158 (42.4)	9.25 (± 6.21)	0.004	[− 4.56, − 0.89]
Group 2: ≥ 4 days	215 (57.6)	11.97 (± 11.58)		
Subgroup a: ≥ 4–7 days	154 (41.3)	11.94 (± 11.5)	0.97	[− 3.6, 3.49]
Subgroup b: > 7 days	61 (16.4)	12.02 (± 11.86)		
*Subgroup analysis*				
Group 1: 0–3 days	158 (42.4)	9.25 (± 6.21)	0.011	[− 4.77, − 0.63]
Subgroup a: ≥ 4–7 days	154 (41.3)	11.94 (± 11.5)		
Group1: 0–3 days	158 (42.4)	9.25 (± 6.21)	0.087	[− 5.96, 0.41]
Subgroup b: > 7 days	61 (16.4)	12.02 (± 11.86)		

Unequal variances *t* test

In eyes presenting macula-off status, laser flare value rose significantly the longer the patients’ symptoms lasted, but not in eyes with macula-on status. In the subgroups of eyes with 1, 2, 3 or 4 quadrants affected, we identified no significant difference in laser flare values depending on symptom duration. In phakic eyes, laser flare values were significantly higher with longer symptom duration, but not in pseudophakic eyes. (Table 3)

**Table 3:** Association between laser flare values and anatomical details regarding symptom duration

Macula status (frequency)	Group (frequency)	Laser flare value (mean ± SD) in pc/ms	*p* value	95% Confidence interval
On On	Group 1: 0–3 days (*n = *78) Group 2: ≥ 4 days (*n = *80)	8.06 (± 4.74) 9.51(± 7.9)	0.124	[− 3.32, 0.4]
Off Off	Group 1: 0–3 days (*n = *104) Group 2: ≥ 4 days (*n = *111)	10.41 (± 7.21) 14.27 (± 13.83)	0.013	[− 6.9, − 0.82]
*Extent of RD*				
1 quadrant	Group 1: 0–3 days (*n = *47) Group 2: ≥ 4 days (*n = *53)	6.65 (± 3.87) 6.97 (± 4.3)	0.695	[− 1.94, 1.3]
2 quadrants	Group 1: 0–3 days (*n = *89) Group 2: ≥ 4 days (*n = *108)	9.93 (± 6.7) 10.2 (± 7.5)	0.798	[− 2.3, 1.77]
3 quadrants	Group 1: 0–3 days (*n = *19) Group 2: ≥ 4 days (*n = *36)	10.92 (± 5.77) 13.77 (± 8.73)	0.154	[− 6.8, 1.1]
4 quadrants	Group 1: 0–3 days (*n = *3) Group 2: ≥ 4 days (*n = *18)	18.93 (± 7.75) 33.68 (± 22.19)	0.06	− 30.27, 0.78]
*Lens status*				
Phakia	Group 1: 0–3 days (*n = *82) Group 2: ≥ 4 days (*n = *133)	7.26 (± 4.73) 10.57 (± 11.57)	0.004	[− 5.54, − 1.08]
Pseudophakia	Group 1: 0–3 days (*n* = 76) Group 2: ≥ 4 days (*n = *82)	11.39 (± 6.9) 14.23 (± 11.3)	0.057	− 5.76, 0.08]

Unequal variances *t* test

We conducted a one-way analysis of variance (ANOVA) for 373 eyes to assess a difference in LFP values among different groups in symptom-duration terms (group 1 *n* = 158, subgroup a *n* = 154 and subgroup b *n* = 61). As there was no homogeneity of variance (Levene’s test, *p* < 0.001) Welch’s ANOVA was performed. We observed a significant difference in LFP values among symptom-duration groups (Welch’s F (2, 141.36) = 4.23, *p* = 0.016). Games-Howell post hoc analysis revealed a significant difference between LFP values in group 1 (0–3 days) and subgroup a (≥ 4–7 days) (*p* < 0.029, MDiff = − 2.7, 95%-CI [− 5.17, − 0.22]).

Multiple linear regression was conducted to determine the relative contribution of each independent variable. We included a total of 367 eyes in multiple linear regression to determine the relative contribution of independent variables. We excluded 6 eyes with studentized deleted residuals (SDR) over 3. The model had no autocorrelation as the value of the Durbin-Watson statistic was 1.5. The *R*^2^ for the overall model was 0.26 (adjusted *R*^2^ = 0.25). Lens status (*p* < 0.001) and extent of RD (*p* < 0.001) were statistically significant coefficients.

## Discussion

Since there seem to be numerous factors influencing LFP, a comprehensive scientific analysis of possible associations is desirable. Few studies have investigated LFP in eyes with RRD [3, 4, 10–12], and to date no data has been published describing the potential associations between laser flare values, anatomical details, and symptom duration in RRD. We investigated these characteristics to enhance existing knowledge and as experience has shown that they are often associated with each other in everyday clinical practice in the care of patients with retinal detachment.

In the current study, we found that a longer symptom duration in eyes with primary RRD in the first week leads to an increase in laser flare values potentially because of the persisting disruption in the blood-ocular barrier [7]. This is supported by the fact that eyes with longer-lasting RRD and higher laser flare values reveal greater inflammation and infiltration by activated macrophages over time. Together with the neurosensory retina’s being separated from the retinal pigment epithelium, this is associated with changes in cytokine and chemokine levels [2, 5, 6, 13].

Furthermore, there seems to be a relationship between anatomical features in case of RRD, such as the macula attachment status and quadrant numbers affected by the RRD. We demonstrate that these characteristics are also associated (in terms of their extent) with significantly higher laser flare values. Moreover, in eyes with advanced RRD (macula off, 4 affected quadrants) we noted a significant association with aqueous flare intensity and longer symptom duration. These results confirm the analyses from previous studies and appear reasonable in the light of the previously described clinical understanding of blood-ocular barrier disruption in patients with retinal detachment.

Interestingly, pseudophakic eyes revealed higher laser flare values than phakic eyes. However, as mentioned before in phakic eyes, laser flare values were significantly higher with longer symptom duration, but not in pseudophakic eyes. A possible cause of increased readings in pseudophakia could be an increased flare due to recent cataract surgery. Unfortunately, prior surgeries are often performed outside of our clinic in an outpatient setting and complete data regarding the exact time interval between other procedures such as cataract surgery are not always available. In addition, it must be considered that in pseudophakia there might be an increased flow of protein from the vitreous cavity into the anterior chamber of the eye. In previous studies, Miyake's analyses using vitreous fluorophotometry indicated that blood-ocular barrier disruption was related to surgical method (intracapsular cataract extraction and extracapsular cataract extraction) and age. Furthermore, a longer duration of the barrier disturbance than initially suspected was observed [8, 9]. Further research and analysis on this phenomenon are already underway in a complementary study (unpublished data).

Limitations of the present study are its retrospective design. We could not report the exact duration of symptoms in hours or single days for all patients, so that a detailed association with laser flare values was not possible, and groups had to be formed based on symptom durations as recorded in the patients’ files.

Furthermore, the postsurgical course of laser flare values in association with anatomical parameters and symptom durations deserves further investigation as well. Our data do not yet allow an investigation of a possible association between preoperatively measured flare values and postoperative outcomes including visual acuity improvement and PVR development, as well as surgical success. Another limitation for the investigation of these questions is the size of the studied collective. A much larger cohort would be needed to obtain reliable results, so a multicentre, prospective, randomized study is desirable. Nevertheless, the presented analyses and new results contribute to previous knowledge and lead to a better understanding of the influencing factors of LFP and blood-ocular barrier disorder. In eyes with RRD, we report an association between laser flare values and symptom duration and the anatomical extent of disease in a large patient cohort for the first time. Therefore, LFP should be investigated in further clinical studies in conjunction with assessing the surgical and functional outcome.
